# Annotations

**Published:** 1904-03-12

**Authors:** 


### Annotations.

### Wanted—Ten Thousand a Year.

At the seventh annual meeting of the King's Hospital Fund the Prince of Wales intimated that it was originally hoped to raise the income of this Fund from endowments to £50,000 a year. The assured income of the King's Fund from investments and other permanent sources amounts at present to about £36,000 a year, which is £14,000 a year less than the fixed income from endowments, which it is the aim of the Fund to secure as a minimum. The Prince read a letter from a friend offering to anticipate a provision in his will, and to hand over at once to the Fund by way of gift securities representing the capital sum required to provide one-third of this £14,000 a year, on condition that the remaining two-thirds, *i.e.* £9,332 per annum, is provided by others before December 31st, 1904. The offer has no other conditions attached to it, but the donor intimates his wish that the King's Fund may prevent any portion of the funds subscribed for the relief of the sick poor being diverted to the purposes of medical education. The Prince's friend has thus offered to give something like £150,000 in securities, and the Prince has personally invoked the support of those who are in a position to follow the example of this anonymous benefactor, and so enable the Fund to secure the full results of his generous proposal. What prospects are there that this offer will meet with a full response? The times are no doubt bad, but that cannot have any material effect on the resources of the wealthy, who are in a position to co operate in this splendid effort on behalf of the sick. Already the investments of King Edward's Hospital Fund amount to upwards of £650,000, derived mainly from the generous gifts of the wealthy, and we certainly hope that this £10,000 a year may be forthcoming by way of gift in securities representing the capital sum required. Rich men and women owe as much to the hospitals as the poor, the privilege of personal service in the cause of the sick in the days of health is becoming recognised amongst all classes of Londoners, the King and the Prince of Wales having set everybody an example by their loyal and continuous service in the cause of the hospitals, and it is probable, in our judgment, that the Prince's appeal in support of the offer of his generous friend will result in the income from endowments of the King's Fund being increased to £50,000 a year by the 31st December next. No doubt the objection raised by the anonymous donor to the diversion of funds subscribed for the relief of the sick poor to the purposes of medical education will be welcomed by all classes of hospital supporters. It shows that the Prince's friend is sound in his views of hospital administration, and his objection will add force to the generous offer which he has made.

### The Dispensing of Poisons.

There can be no doubt that the legal restrictions which govern the sale of poisons have acted to the public advantage. They interfere, at least to some extent, with the ready purchase of poison, and they do something to secure an aid to the identity of the purchaser in the event of mischief ensuing. Further, by confining the sale of poisons to pharmacists who have an expert knowledge of their dangerous properties, they introduce another barrier against misadventure and crime. During the last few years the Pharmaceutical Society, with the approval of the Privy Council, have introduced regulations affecting the methods of keeping and dispensing poisons, and these regulations are now enforced on all pharmacists under the risk of heavy penalties. They are, in short, part of the law of the land. In brief, it may be said these regulations demand that each poison shall be stored in such a manner as to be readily distinguishable from other non-poisonous articles. This may be effected by keeping the poisons in a special cupboard and under lock and key, or by the use of some peculiar form of bottle distinguishable by touch from other bottles, or again by the provision of stoppers which are locked or fastened by some other mechanical device. Thus the dispenser can hardly use a poison "by accident." No one will question the wisdom of these regulations. What we now wish to suggest is that they are needed in a private or hospital dispensary not less than in an open pharmacy. The Pharmaceutical Society has no control over either of the two former, but we think both the private practitioner who dispenses his medicines and the authorities of hospitals would be well advised in their own as well as in the public interest voluntarily to adopt the regulations which are compulsory on the pharmacist. Neglect to do so increases the risk of accident and may reasonably, therefore, be the subject of adverse comment, especially in any instance where the risk becomes a reality.

### Training Ships for Pauper Children.

At the Poor-law Conference Mr. Geoffrey Drage, M.P., put in a strong plea for the sending of pauper children to the training ship *Exmouth*. Some guardians do send the boys under their care to the ship, but more would do it if they better understood the benefit of the training given. A good education, a healthy life, and finally an honourable career, are not so frequently available for the wards of the State that any opportunity of providing these should be ignored. When one considers what pauper children are when they enter a workhouse, and what a training such as that which is provided in the *Exmouth* will do for them, one feels that it is a pity that the demand for it is not greater even than it is. Both the navy and the mercantile marine want smart sailors, well trained in their work, sober and reliable. In the navy at least the prospects both of pay and pension are good, and the separation from home and friends, which might act as a deterrent to other boys, can hardly affect these, who are already separated from their families, and are often better so removed. The great danger with pauper children always is that when their education is finished old acquaintances and disreputable kinsfolk will get hold of them and degrade them again to the level from which they have been lifted. For such a sea-faring life is an admirable one, serving the purpose of removing them from evil associations almost as well as emigration, and not depriving the Mother Country of the services of children whom she has brought up.

